# COSMIC: somatic cancer genetics at high-resolution

**DOI:** 10.1093/nar/gkw1121

**Published:** 2016-11-29

**Authors:** Simon A. Forbes, David Beare, Harry Boutselakis, Sally Bamford, Nidhi Bindal, John Tate, Charlotte G. Cole, Sari Ward, Elisabeth Dawson, Laura Ponting, Raymund Stefancsik, Bhavana Harsha, Chai Yin Kok, Mingming Jia, Harry Jubb, Zbyslaw Sondka, Sam Thompson, Tisham De, Peter J. Campbell

**Affiliations:** Wellcome Trust Sanger Institute, Wellcome Trust Genome Campus, Hinxton, Cambridge, CB10 1SA, UK

## Abstract

COSMIC, the Catalogue of Somatic Mutations in Cancer (http://cancer.sanger.ac.uk) is a high-resolution resource for exploring targets and trends in the genetics of human cancer. Currently the broadest database of mutations in cancer, the information in COSMIC is curated by expert scientists, primarily by scrutinizing large numbers of scientific publications. Over 4 million coding mutations are described in v78 (September 2016), combining genome-wide sequencing results from 28 366 tumours with complete manual curation of 23 489 individual publications focused on 186 key genes and 286 key fusion pairs across all cancers. Molecular profiling of large tumour numbers has also allowed the annotation of more than 13 million non-coding mutations, 18 029 gene fusions, 187 429 genome rearrangements, 1 271 436 abnormal copy number segments, 9 175 462 abnormal expression variants and 7 879 142 differentially methylated CpG dinucleotides. COSMIC now details the genetics of drug resistance, novel somatic gene mutations which allow a tumour to evade therapeutic cancer drugs. Focusing initially on highly characterized drugs and genes, COSMIC v78 contains wide resistance mutation profiles across 20 drugs, detailing the recurrence of 301 unique resistance alleles across 1934 drug-resistant tumours. All information from the COSMIC database is available freely on the COSMIC website.

## INTRODUCTION

A large proportion of human cancer is caused by the acquisition of somatic mutations across an individual's lifetime, and large-scale sequencing of patient cohorts has now described millions of such mutations across the human genome. The Catalogue of Somatic Mutations in Cancer (COSMIC) is a database system that collects these somatic mutation data from a variety of public sources into one standardized repository, and make it easily explorable in a variety of graphical, tabulated and downloadable ways. To provide the greatest support in cancer research, COSMIC encompasses all forms of human cancer, from the most frequent cancers in lung, breast and colon, to extremely rare forms of blood cancer, observed by a clinician only once or twice in a career.

Begun in 2004 with curations across only four human genes (1) COSMIC has grown into a large genome-wide system to explore patterns of somatic mutations in all cancers; substantial genetic data are now generated routinely across human tumours and this is captured by expert, standardized curation procedures. Additionally, recent studies have characterized particular mutations in the evolution of genetic resistance to clinical therapeutics. While ensuring that COSMIC encompasses the full coverage of human cancer genetics, these resistance mutations are emphasised in a new section to highlight their impact in clinical oncology.

## DATABASE CONTENT

As described previously (2,3), curation of somatic mutation data into COSMIC proceeds via two parallel pathways. Expert manual literature curation addresses the most important cancer genes, emphasizing full and exhaustive curation of existing literature before release, followed by regular updates. These key cancer genes are selected from the Cancer Gene Census (4), a listing of over 600 genes with substantial evidence describing their strong role in oncology. High quality control results in the rejection of over 30% of papers due to inconsistency or insufficient detail. In parallel, expert curation of genome-wide tumour analyses requires manual assignment of tumour classifications and clinical details, but large files of genetic variant data are annotated and uploaded via a semi-automated system using Ensembl as a source of transcriptome data. Total contents in the v78 release (Sept 2016) are described in Table 1.

**Table 1.** Total contents in version 78 of the COSMIC database (September 2016)

**1 235 846**	Tumour Samples
**4 067 689**	Observed Coding Mutations
**18 029**	Observed Gene Fusions
**1 271 436**	Copy Number Variants
**9 175 462**	Gene Expression Variants
**7 879 142**	Differentially Methylated CpGs
**13 347 517**	Non-coding Variants
**187 429**	Structural Mutations
**23 096**	Papers: Manual Curation
**393**	Genomic Publications
**277**	TCGA/ICGC/Cell Line Studies
**28 366**	Whole Genomes

Somatic mutation data are collected across all cancer diseases, currently 1335 disease descriptions across more than 5000 detailed classifications. Manual literature curation focuses on point mutations (single-nucleotide mutations, small insertions and deletions) and gene fusions. However, genome-wide tumour profiling can be much broader. While genomic literature usually emphasizes point mutations, larger consortium-focused data portals including The Cancer Genome Atlas (5) (TCGA; http://cancergenome.nih.gov) and International Cancer genome Consortium (6) (ICGC; https://dcc.icgc.org) encompass much wider annotations, including point mutations, copy number aberrations, gene expression variants, DNA methylation variants and structural genomic rearrangements, all of which are curated into COSMIC and combined with other curations. Emphasizing the effectiveness of the literature curation approach, over 60% of COSMIC's genome-wide content is curated from scientific literature, whilst less than a third is from consortium sources.

Once curated into COSMIC, all data are standardized and combined in a single database. Every mutation is assigned a co-ordinate on the human reference genome. Since 2015, the default reference is GRCh38, but an archive system on GRCh37 is maintained. Each mutation is either assigned a coding or non-coding annotation. Non-coding descriptions are simple statements of DNA sequence change at specified genomic locations. Coding mutations are additionally annotated according to their impact on the gene they affect, describing coding nucleotide sequence change and peptide sequence change. Manually curated mutations, on key Cancer Census genes, are annotated to transcripts which are most supported in the literature (to best support the community scrutinizing these genes). However, all other gene variations are annotated via the Ensembl database (7) to the longest CCDS transcript (Consensus Coding Domain Sequences; 8). Gene Fusions are described in terms of their exon content from each partner gene. Again, the transcripts that are selected to describe fusions are those most used in the literature, ensuring best support to this community. Gene expression variants are additional numeric values (Z-scores) linked to named genes in COSMIC, referencing expression differences in each sample from a population norm (calculated per-disease cohort). Copy Number changes are annotated to describe a ploidy value for each gene across each tested tumour sample, and described as gain or loss when compared to the average ploidy value across each tumour. Hyper/hypo-methylated CpG dinucleotides are described in a sample when the beta-value for each probe differs substantially from the per-disease population norm. Finally, structural breakpoints are given basic annotations to reference genome co-ordinates, and interpreted into more descriptive consequence annotations when supported by additional published information.

Tumour classification is standardized and described in several nomenclatures to ensure high-resolution, but also to facilitate integrative analyses. Each tumour sample is primarily classified in COSMIC according to a custom vocabulary developed by a team of leading pathologists; available and described here: http://cancer.sanger.ac.uk/cosmic/classification. This classification is now translated into the National Cancer Institute thesaurus ("NCIt"; 9; https://ncit.nci.nih.gov/ncitbrowser), selected as the highest-resolution public ontology across cancer diseases. In parallel, the NCIt classifications are also translated into Experimental Factor Ontology classifications ("EFO"; 10) supporting multiple ways to integrate COSMIC data into wider analyses across cancer genetics.

Increased genomic curation has led to a larger range of variants annotated across the genome, with every human gene now showing a spread of somatic mutation across its length. In order to reduce the complexity of these data and support studies identifying disease drivers, all point mutations in COSMIC is tagged with a single nucleotide polymorphism (SNP) status and a pathogenicity value. The SNP status defines whether each variant has been previously described in the 1000 genomes study (11), or in a panel of normal samples used as controls in ICGC genomic profiling experiments. Pathogenicity scores are determined by the algorithm FATHMM-MKL (12) with scores interpreted to specify each variant as ‘pathogenic’ or ‘neutral’.

A new topic for curation was introduced in 2016, encompassing the genetics of drug resistance. As described, millions of mutations have been observed across cancer, with thousands of alleles implicated in disease causation. Of these, a small number are additionally described as causing clinical resistance to pharmaceutical therapies. These details, when published, are also captured in the COSMIC curation processes, with the latest release (v78; Sept 2016) describing the range of mutations conferring resistance to 20 anti-cancer treatments.

## DATA ACCESS

COSMIC is most easily explored using its public website (http://cancer.sanger.ac.uk). This has been custom-built to make the many annotations in COSMIC easy to explore in user-friendly graphical ways, whilst also providing large tabulated data sets which underpin each presentation. Beyond this website, described below, opportunities have been pursued to make COSMIC available to other resources enabling them to add broad cancer context to further public genomic resources. For instance, COSMIC directly supports pediatric cancer research with full data sets in St. Jude ProteinPaint (13) (https://pecan.stjude.org/#/proteinpaint), provides genome-wide context of cancer somatic variation in Ensembl (7) (http://www.ensembl.org) and highlights oncology targets across the Cancer Gene Census in OpenTargets (https://www.opentargets.org). A COSMIC beacon (http://cancer.sanger.ac.uk/cosmic/beacon) is also available, conforming to GA4GH standards (http://ga4gh.org/#/beacon), allowing COSMIC to be queried within large federated analyses. Full database contents in a variety of useful formats, including Oracle dump files, can be obtained via download (http://cancer.sanger.ac.uk/cosmic/download), requiring registration (https://cancer.sanger.ac.uk/cosmic/register).

### Website overview

**Figure 1.**
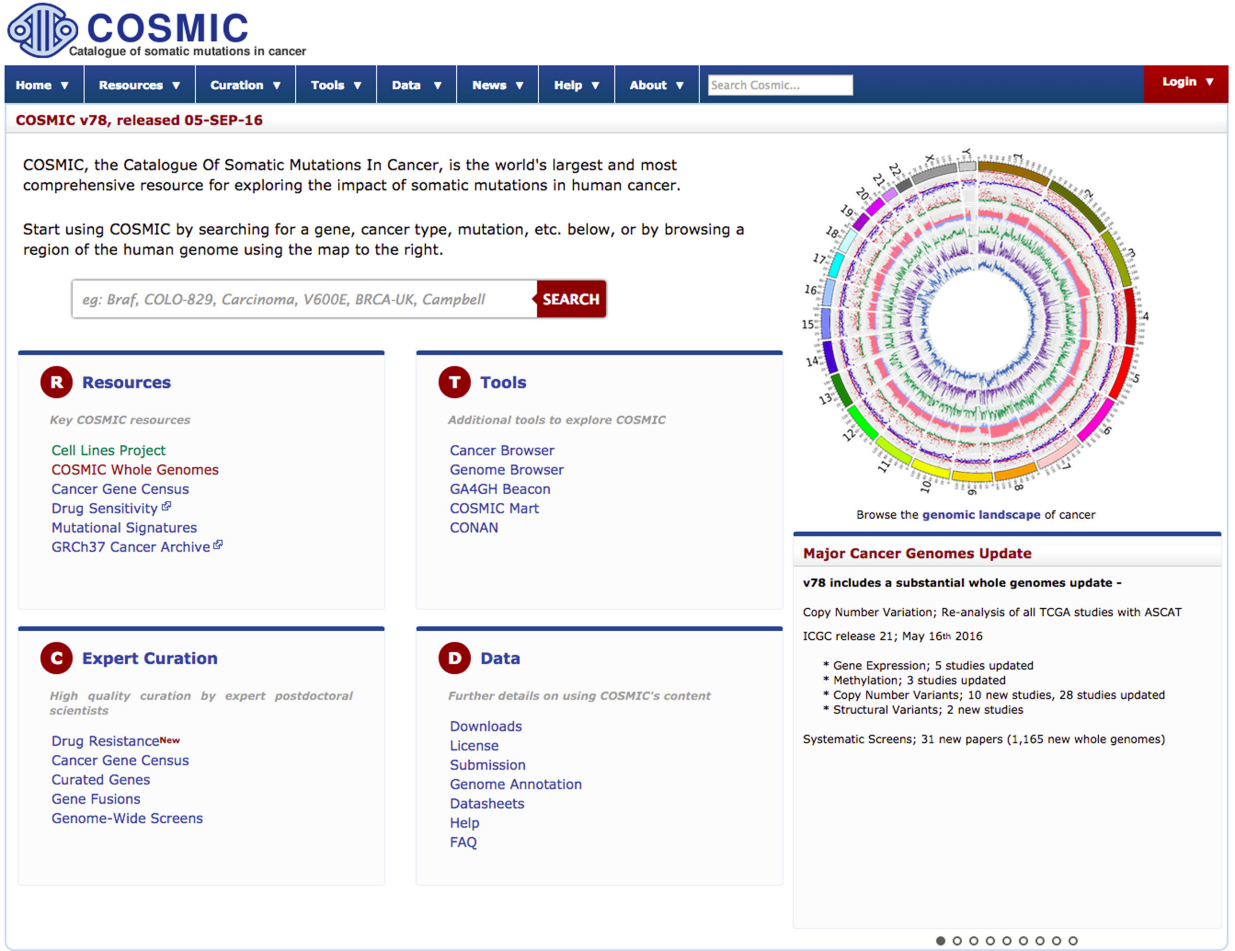
The front page of COSMIC provides easy ways to search and navigate the database; various tools are available for different perspectives on similar data, and multiple descriptive pages detail data curation procedures and current contents.

The COSMIC website is available online at http://cancer.sanger.ac.uk. The front page (Figure 1) offers multiple ways to explore the database (‘Resources’, ‘Tools’), and a range of pages describing the database content, how the data were generated, and details on its access (‘Expert Curation’, ‘Data’). On the right-hand side, a Circos diagram summarizes mutation recurrence across all tumour genomes in COSMIC (version 78, Sept 2016, contains 28,366 genomes and exomes). With a zoomed version for closer exploration (http://cancer.sanger.ac.uk/cosmic/landscape) of recurrent peaks, this image is clickable and responds with a detailed genomic perspective across the region chosen. Primarily, however, COSMIC is navigated via the central ‘Search’ box that will accept any gene name or synonym, mutation syntax, sample/cell line name or paper author and respond with a list of options matching the input search term alongside a few details to aid selection. Once a selection is clicked, a summary page will be shown, overviewing the gene, sample, mutation or publication selected, with further links to specialize a query across COSMIC; in this way somatic mutation trends can be explored in detail across multiple genes and disease types.

Mutation trends across COSMIC are most easily explored when starting with a specific gene or cancer disease. Primarily driven by Human Genome Organisation (HUGO) gene names (http://www.genenames.org/), gene searches will also search all gene synonyms (as well as other terms, including sample/mutation/author) and return any entries that include the characters specified. For instance, searching for ‘PTEN’ will return one gene, since this gene name is unique. However, searching for ‘RAS’ returns 70 entries, since this term occurs in multiple gene names and synonyms; choosing a RAS family member (e.g. KRAS) returns a more specific result.

**Figure 2.**
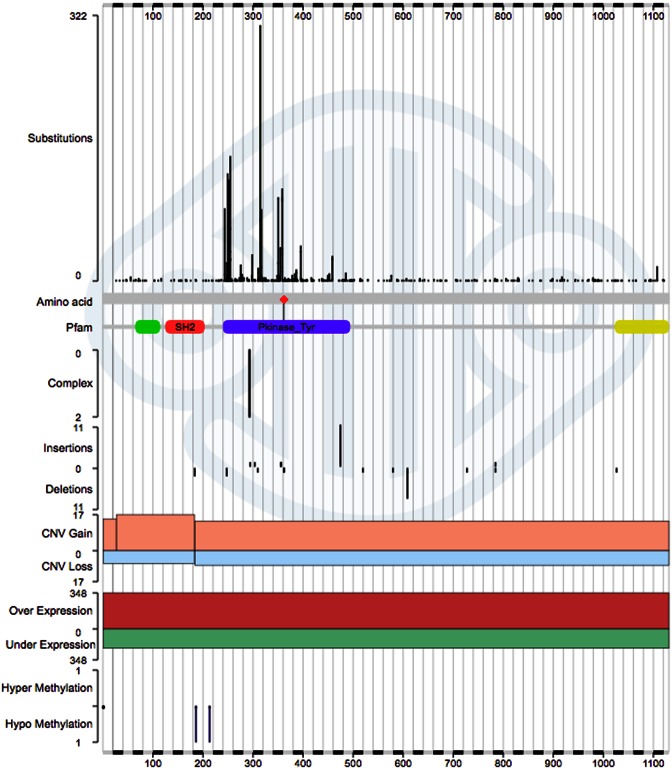
The main Gene Analysis histogram summarises all mutation content across a single gene, in this case gene ABL1. From top-to-bottom, histograms represent mutation recurrence, at each nucleotide/amino acid position, divided into separate segments for single nucleotide substitutions, multinucleotide substitutions, insertions, deletions, copy number gain/loss, gene over/under-expression and CpG hyper/hypo-methylation. Mutation recurrence across the tyrosine kinase domain (highlighted in purple) indicates that this, when mutated, is the key region driving cancer.

Once a gene is selected, COSMIC begins gene analysis by showing the distribution of mutations across the gene's length in a histogram format. In this graphic, the x-axis represents the linear peptide sequence (from Methionine to Stop), with an option to represent nucleotides instead of amino acids (in the right-hand-side Filters box). The histogram graphic summarizes mutation content across several different mechanisms, encompassing several Y axes, vertically aligned, with peak height representing the number of samples in which each mutation was observed (scale bar on left side shows numbers of samples mutated at each position). From top to bottom, mini-histograms show mutation recurrence peaks across the gene sequence for: single nucleotide substitutions, multinucleotide substitutions, small insertions, small deletions, copy number gain/loss, gene overexpression/underexpression and hyper/hypo methylated CpG dinucleotides. For instance, Figure 2 shows the mutation profile for the ABL1 gene, a major driver of blood cancers. Clearly most cancer mutations cluster within and around the tyrosine kinase domain of this peptide, a characteristic of driver mutations in this broad gene family. Using the left-hand scale bars to judge the impact of each mutation type, ABL1 primarily drives cancer development by kinase-domain point mutations and over-expression. Only small numbers of other mutation types are observed. The graphic is zoomable, requiring a simple click-and-drag of the mouse cursor across the region of interest to display it in more detail.

The right-hand side Filters box allows this histogram (together with all information in other tabs on this page) to be sifted and explored in multiple ways, with options to select mutations from a particular tissue or disease, or a particular gene region, specify only confirmed somatic mutations, or only cell lines or patient samples, as well as a variety of other more mechanistic mutation characteristics.

The webpage shows different perspectives across a gene's mutation content in separate ‘tabs’, arranged at the top of the page, offering several ways to scrutinize the large amount of information available. Particularly useful to explore the impact of selected genes across hundreds of cancer types curated, ‘Tissue’ shows a breakdown of mutations across tissues and diseases arranged in a matrix. Four mutation types are shown on the x-axis, and primary tissue types on the y-axis. Between these, small red single-bar histograms show the impact of each mutation type on each tissue, larger bars indicating a higher impact, allowing very rapid evaluation of which tissues are affected most by the gene selected (‘n’ is also shown to evaluate the significance of the red bar; if the number is small, the significance is low). For ABL1, the longest bar is ‘Haematopoietic & Lymphoid’, highlighting its impact in blood cancers. Clicking on the phrase ‘Haematopoietic & Lymphoid’ will show a breakdown of the cancer disease classifications under this tissue, indicating that ABL1 mutations primarily drive 28% of evaluated Chronic Myeloid Leukaemias, and 32% of Acute Lymphoblastic Leukaemias (in COSMIC v78).

### Cancer browser

Cancer diseases are most easily navigated using the Cancer Browser (http://cancer.sanger.ac.uk/cosmic/browse/tissue), where a selection can be made from a list, avoiding the need to type often complex pathology terms. Selection of a primary tissue is essential, then a choice is offered across related subtissue, histology and subhistology terms. These are all clickable, and all choices may be skipped using the top ‘include all’ option. Once a selection is made, clicking ‘Go’ will calculate mutation profiles across all genes for the disease selected. For instance, choosing simply ‘Lung’ cancer and pressing Go, immediately shows the most frequently mutated top 20 genes in Lung cancer (ordered by mutation frequency), primarily highlighting well-characterized genes such as TP53, EGFR and KRAS). Similar to the Gene Analysis pages, additional tabs offer different perspectives on the mutation burden of the selected disease.

### Genome browser

Somatic mutations in cancer are now annotated across the entire human genome. With over 4 million coding point mutations and over 13 million non-coding point mutations, a method to evaluate the genomic context across this information is essential. The COSMIC Genome Browser (http://cancer.sanger.ac.uk/cosmic/browse/genome) achieves this, showing all COSMIC mutation data aligned with a range of genomic annotations, using an instance of the Jbrowse platform (14) that makes enormous data easily and rapidly explorable. The front page to this system offers Search functionality, described above, but returns a genomic (rather than genic) view on the selection made. Initial views show gene structure, point mutations and genome structure breakpoints for the chosen region. Zooming in and out allows exploration of details and trends across a genomic region, with mutation details transforming into histograms when the data becomes too crowded onscreen. On the left side of the screen, a number of additional tracks are offered, divided into annotations of genomic structure/function from the Ensembl database (7) and multiple annotations of somatic mutation from the COSMIC database. These allow the investigation of all somatic mutation and variation data in alignment with structures of coding genes, non-coding RNAs (including microRNAs) and regulatory regions. In addition, the content of dbSNP (15) (https://www.ncbi.nlm.nih.gov/snp/) is made available, to allow the exploration of relationships between somatic mutations in COSMIC and wider human variation encompassed by dbSNP.

### Genetics of drug resistance

**Figure 3.**
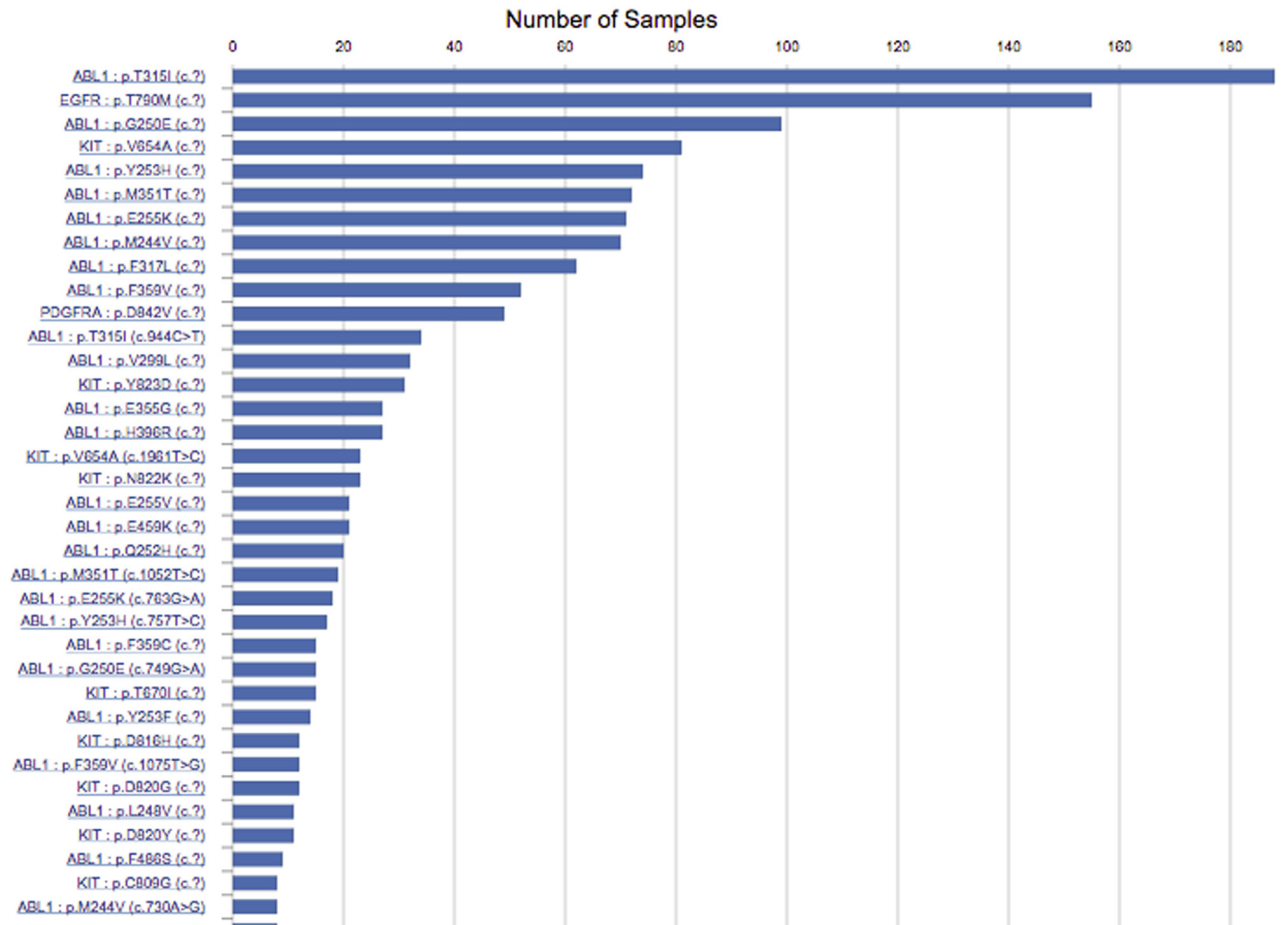
Several named therapeutics have been described treating ABL1-driven cancers, and many studies have reported novel mutations causing resistance to these therapies. This histogram shows a long list of mutations associated with resistance to named therapeutics, with a histogram bar showing their recurrence.

As precision medicine progresses, an increasing literature is emerging which describes genetic responses to targeted therapies. Somatic mutations acquired during tumour evolution are often under selective pressure when mono-therapies are applied to cancer patients, leading to emergence of new clones containing resistant alleles and causing therapeutic failure. COSMIC now curates this information (overviewed at http://cancer.sanger.ac.uk/cosmic/drug_resistance), describing the range of somatic mutations causing resistance to selected therapeutics. In v78 of COSMIC (September 2016), 20 drugs are detailed, describing 301 unique sequence variants that have caused drug resistance in 1934 tumour samples. This information is presented in the COSMIC website in graphical form with pie charts and histograms describing the recurrence of genes and mutations in the evolution of therapeutic resistance. For example, tumours driven by ABL1 mutations have been treated with four named therapies (Imatinib, Dasatinib, Bosutinib and Nilotinib) as well as other unnamed kinase inhibitors. The landscape of resistance to these drugs is presented at http://cancer.sanger.ac.uk/cosmic/gene/analysis?ln=ABL1#dt, with options to see the range of genes involved, or the much larger range of sequence variants (Figure 3).

### COSMIC cell lines

In parallel to the COSMIC curations shown on the main COSMIC website, ‘COSMIC Cell Lines’ (http://cancer.sanger.ac.uk/cell_lines) makes available the molecular profiling of 1015 cancer cell lines commonly used in laboratory research, particularly in evaluations of pharmaceutical activity and efficacy. This is a separate but parallel database, overviewing updates across the information published in (16), including full exome sequencing of each cell line, copy number analysis and gene expression analysis. Additional information is added as further experiments are completed. The data are hosted in an analytic website with identical functionality to the curated COSMIC system (described above), with additional links to raw data files and drug sensitivity evaluations (hosted at http://www.cancerrxgene.org/).

### Downloads

In addition to the free and open presentation of COSMIC in its public website, COSMIC is also available for download in multiple formats (https://cancer.sanger.ac.uk/cosmic/download). Multiple files in TSV (Tab-separated format) or VCF (Variant call format) formats provide complete data sets across each COSMIC release, and the entire database is provided as a full Oracle export file. Downloading complete COSMIC data sets requires registration, but subsequent access is full and free to all academic and non-profit institutes (https://cancer.sanger.ac.uk/cosmic/register).

## FUTURE WORK

**Figure 4.**
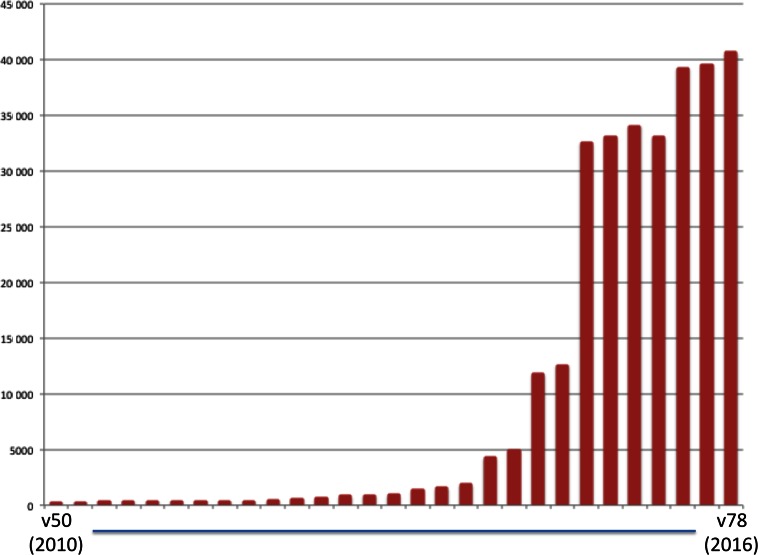
COSMIC has grown rapidly in recent years, as larger numbers of genes are curated, and additional mutation mechanisms are encompassed. The graphic shows a simple representation of the size of the database supporting the COSMIC website from 2010 to 2016 (gzipped Oracle dmp file, in Mb).

COSMIC is built primarily via curation of published literature by expert scientists. This is still a major focus, and as this team expands, COSMIC has been able to encompass greater breadth of annotations, recently resulting in the new focus on Genetics of Drug Resistance. All aspects of cancer genetic curation will continue, with an emphasis on gathering details for deep descriptive annotations across key cancer genes, and broad molecular profiles across cancer genomes. The COSMIC database has grown considerably over the last few releases, and it is anticipated this growth will continue (Figure 4).

The information in the COSMIC database is made available in a number of different ways, and these will be expanded. By encompassing analytic navigation of large data across multiple diseases and mechanisms, the COSMIC website is increasingly complex, and redesigning some of its presentations will make it easier to explore. Supporting external integration of COSMIC is also a priority. A programmatic API will overcome much of the current need to download large data files, and will be very helpful in supporting studies needing to bring large data sets together. In these ways, COSMIC will continue to support a wide range of cancer research investigations, from those exploring primary tumour genetics, through diagnostic and pharmaceutical target identification, to those seeking to understand responses to clinical intervention.
